# Atomic-Scale Investigation of Deformation Behavior and Dislocation Evolution During Metal Spinning Based on Molecular Dynamics Simulations

**DOI:** 10.3390/mi17070772

**Published:** 2026-06-25

**Authors:** Piyao Liu, Linsen Song, Ziwei Jiang, Zhenhui Li, Wei Liang, Xuanda He

**Affiliations:** School of Mechanical and Electrical Engineering, Changchun University of Science and Technology, Changchun 130022, China; 2023200116@mails.cust.edu.cn (P.L.); 2022200103@mails.cust.edu.cn (Z.J.); lizhenhuicust@sina.com (Z.L.); liang811@cust.edu.cn (W.L.); hexuandacust@sina.com (X.H.)

**Keywords:** metal spinning, molecular dynamics, plastic deformation, dislocation evolution, high-temperature deformation

## Abstract

Localized stress concentration and defect accumulation are prone to occurring during metal spinning because of the coupled effects of complex loading and interfacial friction. In this study, a molecular dynamics model of metal spinning was established to investigate the effects of process parameters and temperature on the mechanical response, material flow, contact loading, and dislocation evolution behavior within the contact zone. The results indicate that the optimal deformation coordination is achieved with an arc radius of 25 Å, an indentation depth of 8 Å, and a tangential velocity of 1.5 Å/ps. Analysis of the normal and tangential forces shows that the normal load is rapidly established during the indentation stage, whereas the tangential load continuously increases with material shear transport. Both loads decrease significantly with increasing temperature. Elevated temperature effectively suppresses dislocation accumulation and simplifies the dislocation structure, causing the plastic deformation behavior to gradually transition toward a dominant primary slip-system mode. This study reveals the local deformation and dislocation evolution mechanisms during spinning and provides theoretical guidance for the process optimization of thin-walled spinning components.

## 1. Introduction

Metal spinning, as a typical localized continuous plastic formation process, has been widely applied in the aerospace, energy equipment, and high-end pressure vessel manufacturing fields [1,2,3]. Compared with conventional machining or welding-based forming methods, spinning offers advantages including lower forming force, higher material utilization, improved dimensional accuracy, and superior mechanical properties, making it particularly suitable for the integrated fabrication of complex thin-walled rotational components [4,5]. However, severe local contact, continuous shear sliding, and complex stress transfer occur between the indenter and workpiece during spinning, which can induce stress concentration, plastic instability, and micro-defect evolution under coupled high-strain and high-strain-rate conditions [6,7]. Under large-deformation, high-feed-rate, or high-speed spinning conditions, non-uniform material flow, wall-thickness fluctuation, and microcrack initiation may further occur, thereby deteriorating formation quality and service performance [8,9,10].

To improve spinning quality and optimize material flow behavior, extensive studies have been conducted on indenter geometry, process parameters, and interfacial contact behavior [11,12,13]. Previous studies demonstrated that parameters such as indenter nose radius, reduction depth, and tangential velocity significantly affect stress distribution, plastic flow behavior, and defect evolution in the contact zone [14]. Appropriate indenter geometries can alleviate local stress concentration, improve material flow continuity, and suppress shear localization [15]. For example, Shen Y et al. [16] reported that a larger indenter nose radius effectively enlarges the contact area and reduces local plastic deformation concentration by lowering the unit-area load. GAO P et al. [17] found, through finite element analysis, that reducing the reduction depth appropriately can improve wall-thickness uniformity and suppress instability defects. Tran M V et al. [18] further demonstrated that tangential velocity strongly influences material flow stability and interfacial friction behavior, while excessive velocity may induce localized high-shear regions and stress fluctuations. Although considerable progress has been achieved in macroscopic investigations of spinning processes, the underlying microscopic mechanisms associated with atomic-scale slip, dislocation evolution, and local structural rearrangement during spinning remain insufficiently understood.

At the microscopic scale, the spinning process is essentially a dynamic evolution process involving atomic compression, shear, slip, and rearrangement under the action of the indenter. Local stress transfer, atomic migration, and the nucleation and propagation of dislocations within the contact zone jointly determine the plastic flow capability of the material and the formation behavior of microscopic defects [19]. Although conventional finite element methods can effectively describe macroscopic stress–strain distributions, they are unable to directly reveal key physical processes such as dislocation evolution, local lattice distortion, and atomic-scale friction behavior [20]. Molecular dynamics (MD) simulations can directly characterize the dynamic response of materials at the atomic scale under complex loading conditions and have been widely applied in nano-indentation, cutting, and plastic deformation studies [21,22,23,24]. Previous studies have demonstrated that MD methods can effectively capture dislocation nucleation, shear-band formation, and local thermal evolution in metallic materials subjected to external loading [25]. For example, Song W et al. [26] revealed the relationship between dislocation propagation and stress release under localized contact conditions through MD simulations. Podd E L et al. [27] pointed out that the plastic flow capability of materials is closely related to the local shear-strain distribution. He Z et al. [28] further found that elevated temperature can promote dislocation annihilation and structural rearrangement, thereby improving the plastic deformation compatibility of materials. Overall, existing studies have mainly focused on nano-indentation or cutting processes, whereas atomic-scale investigations concerning the synergistic effects of indenter geometry, reduction depth, and tangential velocity on material flow, stress transfer, and dislocation evolution during spinning remain relatively limited.

Based on the above background, a three-dimensional molecular dynamics model of the metal spinning process was established in this study to systematically investigate the microscopic deformation behavior within the spinning contact zone under different indenter arc radii, indentation depths, tangential sliding velocities, and temperature conditions. Through comprehensive analyses of Von Mises stress, shear strain, atomic displacement, contact loading, and dislocation evolution behavior, the intrinsic relationships among stress transfer, material flow, interfacial shear effects, and defect evolution during spinning were revealed. Furthermore, the effects of process parameters and temperature on plastic deformation, shear localization, load evolution, and dislocation behavior within the contact zone were clarified at the atomic scale.

## 2. Establishment of the Molecular Dynamics Model

### 2.1. Indenter and Workpiece Models

The workpiece material used in this study was 20MnMo low-alloy structural steel. However, because 20MnMo is a typical multi-component alloy steel system, its actual composition distribution and microstructure are difficult to fully reconstruct at the molecular dynamics scale, particularly for complex features such as pearlite, tempered structures, and alloy-element interfaces [29]. Therefore, the workpiece material was simplified as a homogeneous α-Fe crystal system. Although the present model does not explicitly consider the effects of alloying elements, grain boundaries, or microstructural heterogeneity, α-Fe represents the dominant ferritic matrix phase of 20MnMo steel and can reasonably capture the essential BCC deformation characteristics, including stress-induced slip, plastic flow, and dislocation evolution. Accordingly, the intrinsic deformation mechanisms of the material surface layer in the spinning contact zone were investigated through analyses of atomic migration, stress transfer, and dislocation evolution under coupled compression–shear loading. It should be noted that the present model is intended to reveal the fundamental atomic-scale deformation and defect-evolution mechanisms during spinning rather than quantitatively reproduce the exact microstructural response of the actual alloy.

**Figure 1:**
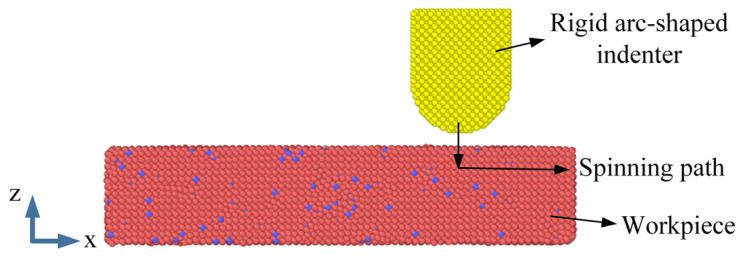
Schematic diagram of the indenter and workpiece model.

To characterize the local contact behavior between the indenter and workpiece during spinning, a rigid indenter–workpiece contact model was established, as shown in Figure 1. The workpiece was constructed using an α-Fe crystal structure and modeled as a cuboid with dimensions of 200 Å × 50 Å × 40 Å to represent the local surface region of the workpiece. The indenter was modeled as a rigid arc-shaped structure and treated as a rigid body to equivalently represent the local contact region of the spinning indenter. Following conventional molecular dynamics simulation procedures, the workpiece was divided into a fixed layer, a thermostat layer, and a Newtonian layer. The fixed layer was used to constrain rigid-body motion of the system, while the thermostat layer employed a Nose–Hoover heat bath to maintain the target temperature and dissipate generated heat. The Newtonian layer served as the primary deformation region without temperature control, thereby ensuring the realistic dynamic response of the material under external loading.

The main simulation parameters of the spinning process are summarized in Table 1. The initial temperature of the simulation system was set to 293 K. Before loading, the system was relaxed for 10,000 steps using a Nose–Hoover thermostat to eliminate initial nonequilibrium effects. The indenter was initially positioned above the workpiece surface with a certain clearance to avoid nonphysical initial contact interactions. During the spinning process, the workpiece was loaded through coupled normal indentation and tangential sliding of the indenter. The normal indentation depths were set to 6 Å, 8 Å, 10 Å, and 12 Å to represent different local loading conditions during formation. Constant tangential sliding velocities of 1 Å/ps, 1.5 Å/ps, 2 Å/ps, and 2.5 Å/ps were applied to simulate the relative motion between the indenter and workpiece. To systematically investigate the influence of indenter geometry on local deformation behavior, the indenter arc radii were set to 15 Å, 20 Å, 25 Å, and 30 Å, respectively. Through these parameter combinations, the effects of indenter size, loading depth, and sliding velocity on material flow, stress distribution, and defect evolution in the local spinning contact zone can be systematically analyzed.

### 2.2. Interatomic Potential Functions

The interatomic potential is a core parameter in molecular dynamics simulations, and its proper selection directly affects the accuracy of atomic interaction descriptions, thereby determining the mechanical and thermal responses of materials during deformation. According to the characteristics of different atomic interactions, the Embedded Atom Method (EAM) potential and Morse potential were employed in this study to describe the simulation system [30,31].

#### 2.2.1. EAM Potential

Because the Embedded Atom Method (EAM) potential can effectively characterize metallic bonding and has been widely validated in molecular dynamics studies of body-centered cubic (BCC) Fe and its alloy systems, the EAM potential was employed in this study to describe the interactions between Fe atoms in the workpiece. Within the EAM framework, the total energy of the system, ETotal, can be calculated according to the formulation reported in Ref. [32]:

(1) $$E_{Total}=\frac{1}{2}\sum_{ij}V\left(r_{ij}\right)+\sum_{i}F\left({\bar{\rho }}_{i}\right)$$

where $V\left(r_{ij}\right)$ is the pair interaction energy between atoms i and j separated by a distance $r_{ij}$, and F is the embedding energy of an atom i as a function of the host electron density ${\bar{\rho }}_{i}$. The latter is given by

(2) $${\bar{\rho }}_{i}=\sum_{j\neq i}\rho \left(r_{ij}\right)$$

where $\rho \left(r_{ij}\right)$ is the electron density function assigned to an atom.

#### 2.2.2. Tersoff Potential

Since no stable chemical bonding exists between the diamond indenter and α-Fe, the Morse potential was employed to describe the interfacial atomic interactions. The Morse potential can effectively characterize short-range repulsion and weak attractive interactions and has been widely used in molecular dynamics simulations of heterogeneous material interfaces. Its expression is given in Equation (3) [33]:

(3) $$E=D_{0}\left[e^{-2\alpha (r_{ij}-r_{0})}-2e^{-\alpha (r_{ij}-r_{0})}\right],\;r_{ij}<r_{0}$$

In this formulation, $D_{0}$ denotes the bond dissociation energy, $r_{0}$ is the equilibrium interatomic distance, and α is a parameter controlling the steepness of the potential well. The corresponding parameter values used in this study are listed in Table 2. The Morse potential parameters used in this study were adopted from Refs. [34,35], where they were previously validated for characterizing the interfacial interactions between the tool and workpiece during contact-deformation simulations.

### 2.3. Experimental Methods

**Figure 2:**
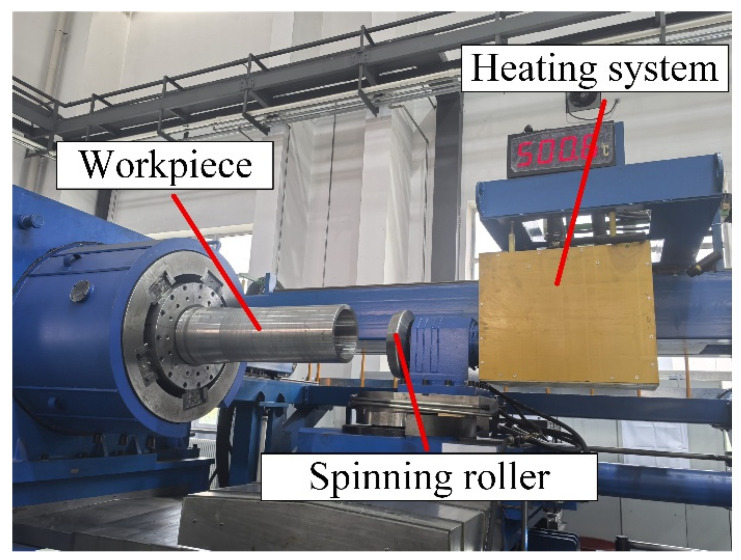
Experimental equipment.

The experiments were conducted on an Rx650 single-wheel hot-spinning machine, and the experimental setup is illustrated in Figure 2. The equipment mainly consists of a spindle clamping system, a roller feeding system, a heating system, and a control system, enabling the continuous hot-spinning formation of cylindrical workpieces under elevated-temperature conditions. The workpiece used in this study was a cylindrical billet, and its dimensional parameters are listed in Table 3. The billet had an outer diameter of Φ310 mm, an inner diameter of Φ295 mm, and a length of 1520 mm. After the spinning process, the formed workpieces were sectioned for sampling, and a laser scanning confocal microscope was employed to characterize the three-dimensional surface morphology and measure the surface roughness.

## 3. Results and Discussion

### 3.1. Mechanical Response of the Spinning Contact Zone

During the spinning process, complex multiaxial stress fields are generated within the contact zone under the coupled effects of normal indentation and tangential sliding of the indenter, resulting in significant local plastic deformation and stress evolution behavior [36]. Because the material simultaneously experiences normal compression and tangential shear, a single stress component cannot fully characterize the actual stress state within the contact zone. Therefore, Von Mises stress and shear strain were adopted as the primary evaluation parameters in this study [37]. Based on distortion energy theory, Von Mises stress can comprehensively reflect the multiaxial stress state inside the material and is widely used to characterize yielding behavior and plastic deformation in metal forming analyses [38]. The calculation expression of Von Mises stress is given in Equation (4) [39].

(4) $$\sigma_{Mises}=\sqrt{\frac{1}{2}{\left(\sigma_{zz}-\sigma_{xx}\right)}^{2}+\frac{1}{2}{\left(\sigma_{xx}-\sigma_{yy}\right)}^{2}+\frac{1}{2}{\left(\sigma_{yy}-\sigma_{zz}\right)}^{2}+3(\tau_{xy}^{2}+\tau_{yz}^{2}+\tau_{zx}^{2})}$$

Shear strain can characterize local slip and shear deformation behavior inside the material during the spinning process, and its distribution can reflect the material flow path and the degree of localized deformation. Analysis of the shear-strain distribution can further reveal the formation and propagation behavior of shear bands and enable evaluation of the material flow characteristics under different process conditions. The calculation expression for shear strain is given in Equation (5) [39].

(5) $$\eta_{i}^{Mises}=\sqrt{\frac{6\eta_{xy}^{2}+6\eta_{yz}^{2}+6\eta_{zx}^{2}+{\left(\eta_{xy}-\eta_{yy}\right)}^{2}+{\left(\eta_{yy}-\eta_{zz}\right)}^{2}+{\left(\eta_{zz}-\eta_{xx}\right)}^{2}}{6}}$$

where $\eta_{xx}$, $\eta_{yy}$, and $\eta_{zz}$ are the normal strain components, and $\eta_{xy}$, $\eta_{yz}$, and $\eta_{zx}$ are the shear-strain components.

**Figure 3:**
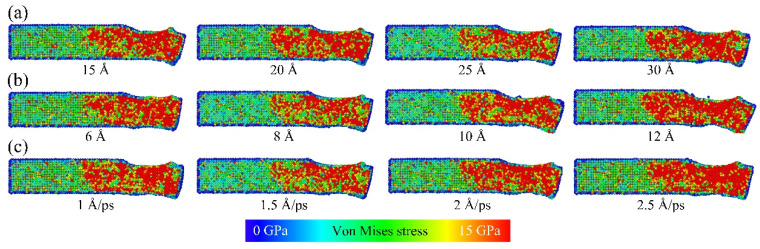
Effects of different process parameters on the Von Mises stress distribution in the local spinning contact zone: (**a**) different arc radii, (**b**) different indentation depths, and (**c**) different tangential sliding velocities.

Figure 3 presents the Von Mises stress distributions in the spinning contact zone under different arc radii, indentation depths, and tangential sliding velocities. Specifically, Figure 3a shows the stress distributions under different arc radii, Figure 3b presents the stress distributions under different indentation depths, and Figure 3c illustrates the stress distributions under different tangential velocities.

As shown in Figure 3a, the high-stress regions are mainly concentrated in the indenter contact zone and the forward material flow region, exhibiting a clear propagation trend along the tangential direction. When the arc radius is 15 Å, obvious local stress concentration appears within the contact zone, accompanied by a relatively large high-stress region, indicating that the smaller arc radius results in a limited contact area and higher local loading. As the arc radius increases to 20 Å and 25 Å, the stress distribution gradually becomes smoother and the high-stress region weakens significantly, suggesting that a larger contact arc can effectively disperse local loads. However, when the arc radius further increases to 30 Å, the stress-affected region expands again, indicating that an excessively large contact arc enhances the overall extrusion effect on the material.

Figure 3b shows that the overall Von Mises stress within the contact zone first decreases and then increases as the indentation depth increases from 6 Å to 12 Å. At an indentation depth of 6 Å, the high-stress region is mainly concentrated near the material surface, accompanied by obvious local stress accumulation. When the indentation depth increases to 8 Å, the stress peak gradually propagates into the material interior, indicating that moderate normal compression can enhance the transfer of contact loading into the subsurface region. However, when the indentation depth further increases to 10 Å and 12 Å, the high-stress region ahead of the contact zone expands again and distinct local stress concentration appears. This suggests that excessive normal compression strengthens the atomic extrusion effect, resulting in continuous stress accumulation near the contact front.

Figure 3c indicates that the tangential velocity has a significant influence on the stress distribution within the contact zone. When the tangential velocity is 1 Å/ps, the overall stress level is relatively low, while the high-stress regions are distributed discretely, indicating limited load transfer capability inside the material under low-sliding-velocity conditions. As the tangential velocity increases to 1.5 Å/ps, the stress peak near the contact front decreases significantly, suggesting that a moderate sliding velocity promotes continuous load release along the material flow direction and alleviates stress accumulation near the contact front. However, when the tangential velocity further increases to 2 Å/ps and 2.5 Å/ps, obvious stress intensification appears again near the contact front. This is mainly because the local load is transported rapidly under high-speed sliding conditions, while atomic rearrangement inside the material cannot respond in time, resulting in pronounced dynamic stress accumulation near the contact front.

**Figure 4:**
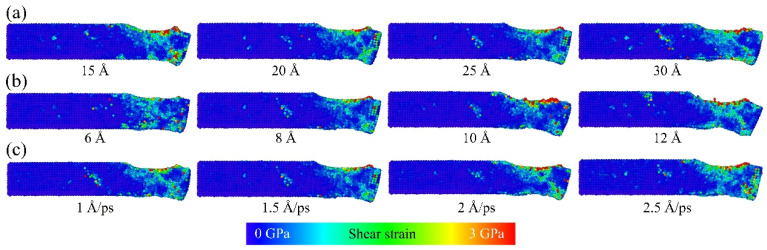
Effects of different process parameters on the shear-strain distribution in the local spinning contact zone: (**a**) different arc radii, (**b**) different indentation depths, and (**c**) different tangential sliding velocities.

Figure 4 presents the shear-strain distributions in the spinning contact zone under different arc radii, indentation depths, and tangential sliding velocities. Specifically, Figure 4a shows the distributions under different arc radii, Figure 4b corresponds to different indentation depths, and Figure 4c presents the distributions under different tangential velocities.

Figure 4a shows that the high-shear-strain regions are mainly concentrated in the indenter contact zone and the forward material flow region, forming distinct shear deformation bands along the material flow direction. When the arc radius is 15 Å, the high-shear-strain region is relatively large and the local shear bands exhibit a discrete distribution pattern. As the arc radius increases to 20 Å and 25 Å, the shear-strain distribution gradually becomes smoother, the shear bands become more continuous, and the localized high-strain regions weaken significantly. Under the 25 Å condition, the shear-strain distribution is the smoothest and the localized shear concentration is the weakest. However, when the arc radius further increases to 30 Å, the affected region of shear strain expands again and discontinuous diffusion appears locally, indicating that an excessively large contact arc enhances the overall plastic traction effect.

In Figure 4b, when the indentation depth is 6 Å, the shear strain is mainly concentrated in the indenter contact region and the forward material flow zone, while the localized high-strain regions are distributed relatively discretely. As the indentation depth increases to 8 Å, the shear-strain distribution gradually becomes smoother, the localized high-strain regions decrease significantly, and the shear deformation progressively propagates into the material interior along the thickness direction. However, when the indentation depth further increases to 10 Å and 12 Å, the shear-strain region expands further along the indenter contact profile and the material flow direction, while obvious local strain accumulation appears near the contact front and outlet region, indicating that excessive normal compression enhances localized shear deformation behavior.

Figure 4c indicates that, when the tangential velocity is 1 Å/ps, the shear strain has already spread over a relatively large region near the contact zone, while the high-shear-strain regions exhibit a broad distribution along the material flow direction and the localized strain intensification remains relatively weak. As the tangential velocity increases to 1.5 Å/ps, the boundaries of the primary shear bands become clearer, and the high-strain regions gradually evolve from broad distributions into concentrated band-like structures, indicating that a moderate sliding velocity improves the propagation efficiency of local shear deformation. When the tangential velocity further increases to 2 Å/ps and 2.5 Å/ps, pronounced high shear-strain intensification gradually appears near the contact front and outlet region, while the shear-band boundaries become significantly strengthened. As a result, the internal shear deformation behavior gradually changes from uniform propagation to localized concentration.

### 3.2. Material Flow Behavior and Control Mechanism

**Figure 5:**
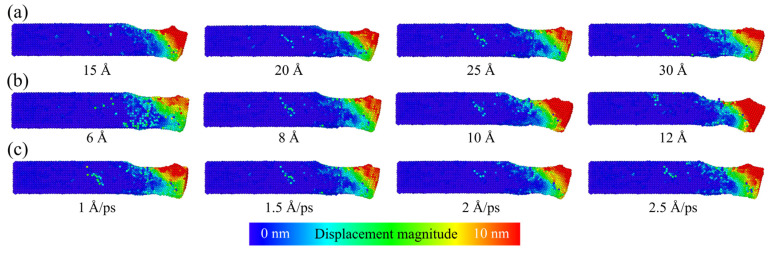
Atomic displacement distributions in the spinning contact zone under different process parameters: (**a**) different arc radii, (**b**) different indentation depths, and (**c**) different tangential sliding velocities.

Figure 5 presents the atomic displacement distributions in the spinning contact zone under different arc radii, indentation depths, and tangential sliding velocities. Specifically, Figure 5a shows the atomic displacement distributions under different arc radii, Figure 5b corresponds to different indentation depths, and Figure 5c presents the distributions under different tangential velocities.

Figure 5a indicates that the high-displacement regions are mainly concentrated in the indenter contact zone and near the material outlet region, exhibiting a clear propagation trend along the material flow direction. When the arc radius is 15 Å, a relatively large high-displacement region has already formed within the contact zone, accompanied by obvious displacement accumulation near the outlet region. As the arc radius increases to 20 Å and 25 Å, the atomic displacement distribution gradually becomes smoother, while the high-displacement region near the outlet weakens significantly and the material flow path along the contact interface becomes more continuous. Under the 25 Å condition, the high-displacement region exhibits the most stable distribution and the weakest local displacement fluctuation. However, when the arc radius further increases to 30 Å, the affected region of shear deformation expands again and discontinuous diffusion appears locally, indicating that an excessively large contact arc disperses material flow and enhances local shear deformation differences.

In Figure 5b, when the indentation depth is 6 Å, the high-displacement region is mainly concentrated near the contact outlet, while the displacement diffusion range remains relatively limited, indicating weak material transport capability under low-normal-compression conditions. As the indentation depth increases to 8 Å, the atomic displacement region gradually expands along the material flow direction and the displacement transition becomes smoother, suggesting that a moderate normal indentation force promotes stable material transport toward the outlet region, while the displacement accumulation near the outlet remains relatively weak. However, when the indentation depth further increases to 10 Å and 12 Å, the high-displacement region expands significantly and a large displacement accumulation region forms near the outlet. This indicates that excessive normal compression enhances the plastic extrusion effect toward the outlet direction, resulting in obvious material accumulation and displacement concentration near the outlet region.

Figure 5c shows that, when the tangential velocity is 1 Å/ps, the high-displacement region is mainly concentrated near the outlet region and the overall displacement level is relatively low, indicating limited atomic migration under low-sliding-velocity conditions. As the tangential velocity increases to 1.5 Å/ps, the displacement distribution becomes more uniform and the high-displacement region expands steadily along the material flow direction, suggesting improved material flow stability. However, when the tangential velocity further increases to 2 Å/ps and 2.5 Å/ps, obvious displacement accumulation appears near the outlet region. Particularly at 2.5 Å/ps, a large high-displacement accumulation zone forms, indicating that excessive sliding velocity enhances local plastic extrusion and aggravates flow non-uniformity.

Based on the combined analyses of the Von Mises stress, shear-strain, and atomic displacement distributions, the condition with an indenter radius of 25 Å, an indentation depth of 8 Å, and a tangential velocity of 1.5 Å/ps exhibited the most favorable deformation characteristics. Under this condition, stress transfer was the most uniform, shear-strain localization was minimized, and material flow remained the most stable. In contrast, lower parameter levels resulted in insufficient stress transfer and discontinuous deformation propagation, whereas higher parameter levels promoted stress concentration, strain accumulation, and displacement localization.

It should be noted that this parameter combination represents only the most favorable reference condition within the investigated parameter range. Its purpose is to reveal the effects of process parameters on atomistic deformation behavior rather than to define the globally optimal parameter set for practical spinning operations.

### 3.3. Contact Load Evolution and Temperature Effects

**Figure 6:**
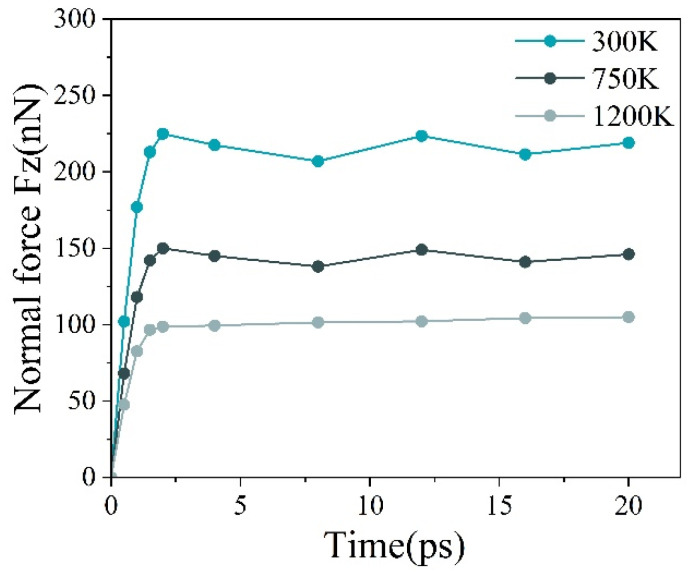
Evolution of the normal force in the spinning contact zone under different temperature conditions.

The optimized process parameters obtained in the previous analysis were an arc radius of 25 Å, an indentation depth of 8 Å, and a tangential velocity of 1.5 Å/ps. Figure 6 presents the evolution of the normal force with time in the spinning contact zone under different temperature conditions. The normal force increases rapidly during the initial stage (0–2 ps), mainly because the indenter first penetrates into the workpiece surface along the normal direction, resulting in a rapid increase in contact area and strong atomic extrusion. As the indenter subsequently enters a stable tangential sliding stage, the normal force gradually stabilizes, with only slight fluctuations. This indicates that a relatively stable plastic flow and load transfer state has been established within the contact zone during steady spinning.

Significant differences in the normal force level can be observed under different temperature conditions. The normal force is the highest at 300 K, remaining at approximately 210–225 nN during the steady stage. At 750 K, the normal force decreases to about 140–150 nN, while it further decreases to around 100 nN at 1200 K. Slight fluctuations can still be observed during the steady stage under all temperature conditions. This behavior is mainly associated with continuous atomic slip, rearrangement, and local structural adjustment within the contact zone during spinning. Although dynamic load fluctuations occur because of variations in atomic migration and plastic flow, the overall force remains relatively stable, indicating good material flow stability under the current process parameters.

**Figure 7:**
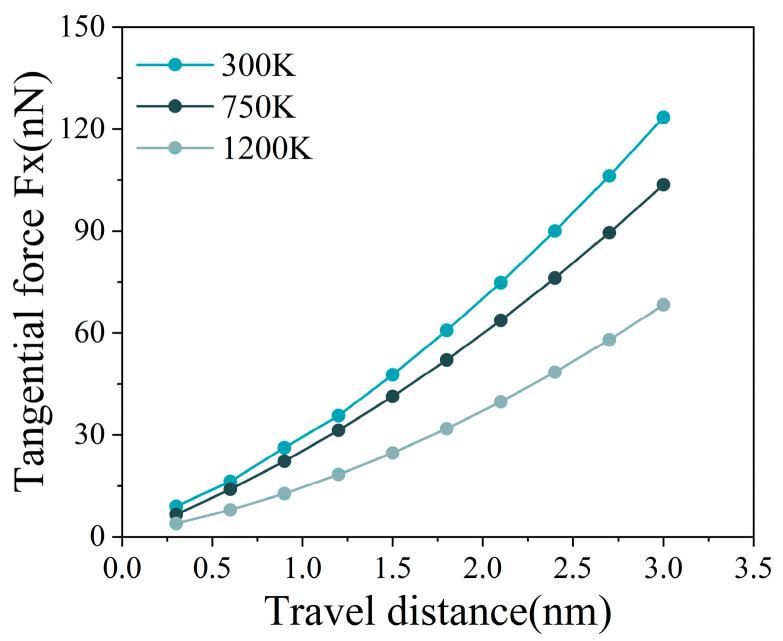
Evolution of the tangential force in the spinning contact zone under different temperature conditions.

Figure 7 presents the evolution of the tangential force with sliding distance in the spinning contact zone under different temperature conditions. As the sliding distance increases, the tangential force continuously increases under all temperature conditions. This is mainly because the material in the contact zone is continuously subjected to shear extrusion during tangential motion of the indenter, causing the plastic deformation region to expand gradually and the interfacial shear resistance to increase continuously. Meanwhile, material accumulation gradually forms ahead of the contact zone as the transport distance increases, further increasing the tangential load.

Significant differences in the tangential force can be observed under different temperature conditions. At 300 K, the tangential force increases most rapidly and exceeds 120 nN at a sliding distance of about 30 nm. At 750 K, the overall tangential force decreases, with a maximum value of approximately 100 nN, while at 1200 K it further decreases to about 70 nN. These results indicate that increasing temperature enhances atomic thermal motion, making it easier for atomic slip and plastic flow to occur and thereby reducing interfacial shear resistance. Under high-temperature conditions, the material softening effect becomes more significant, allowing local stresses in the contact zone to be released more efficiently through atomic migration, which reduces the tangential load required during indenter sliding.

Overall, under the current processing conditions, both the normal force and tangential force exhibit relatively stable evolution characteristics during spinning. The reduction in these forces with increasing temperature is not only attributed to thermal softening and enhanced atomic mobility, but is also closely associated with improvements in stress-transfer behavior and deformation coordination within the contact zone. At elevated temperatures, stress concentration is significantly alleviated, allowing plastic deformation to be distributed more uniformly over a larger region. As a result, material flow becomes smoother and the resistance to deformation decreases. Meanwhile, enhanced stress relaxation weakens local load concentration induced by coupled compression–shear deformation, leading to a more stable load-transfer process and consequently lower normal and tangential forces. These observations are consistent with the results of the Von Mises stress, shear-strain, and atomic displacement analyses presented above, indicating that atomic slip and plastic flow occur more readily at elevated temperatures. This facilitates material flow within the contact zone and reduces local load accumulation during spinning.

### 3.4. Effects of Temperature on Dislocation Evolution and Deformation Behavior

The spinning process is usually accompanied by significant thermo-mechanical coupling effects. Increasing temperature can alter atomic thermal motion, dislocation slip resistance, and stress release behavior, thereby further influencing plastic flow and defect evolution within the material. Based on the optimized process parameters obtained above, the dislocation evolution behavior under 300 K, 750 K, and 1200 K conditions was further compared to reveal the effects of temperature on dislocation nucleation, slip propagation, and defect accumulation, as well as the evolution characteristics of plastic deformation mechanisms under high-temperature conditions.

**Figure 8:**
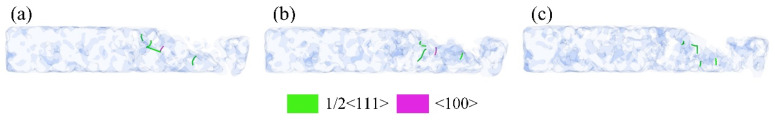
Dislocation evolution characteristics during spinning deformation under different temperature conditions: (**a**) 300 K, (**b**) 750 K, and (**c**) 1200 K.

Figure 8 presents the dislocation morphologies in the spinning contact zone under different temperature conditions. Figure 8a, Figure 8b and Figure 8c correspond to the dislocation evolution results at 300 K, 750 K, and 1200 K, respectively. Under all temperature conditions, 1/2<111> dislocations are dominant. This indicates that the fundamental plastic deformation mechanism of the BCC metal does not change significantly with temperature. Plastic deformation is still mainly accommodated by dislocation slip along the primary slip systems.

At 300 K, the number of dislocations within the contact zone is relatively high. Obvious dislocation accumulation can be observed in local regions, accompanied by certain dislocation interactions and entanglement structures. This indicates that atomic thermal motion is limited under low-temperature conditions. As a result, dislocation slip is more easily hindered by local stress fields, leading to continuous dislocation accumulation and severe lattice distortion in local regions. When the temperature increases to 750 K, the number of dislocations within the contact zone decreases and the local dislocation accumulation becomes weaker. The dislocation distribution gradually changes from localized concentration to a more dispersed state. This indicates that atomic thermal activation is enhanced at intermediate temperature, allowing part of the local stress to be released through dislocation slip and rearrangement. As a result, dislocation accumulation is reduced. The dislocation entanglement structures also decrease significantly, suggesting that the local plastic deformation gradually changes from severe localization to a relatively stable slip-dominated state. When the temperature further increases to 1200 K, the number of dislocations within the contact zone decreases significantly, and only a small number of dispersed dislocation structures remain. Large-scale local dislocation accumulation almost disappears. This indicates that atomic diffusion is greatly enhanced under high-temperature conditions, making it easier for dislocation slip, rearrangement, and annihilation to occur. As a result, dislocation accumulation is significantly reduced. Therefore, it is difficult for high-density dislocation entanglement structures to form at high temperature, leading to weaker lattice distortion and reduced local defect accumulation.

**Figure 9:**
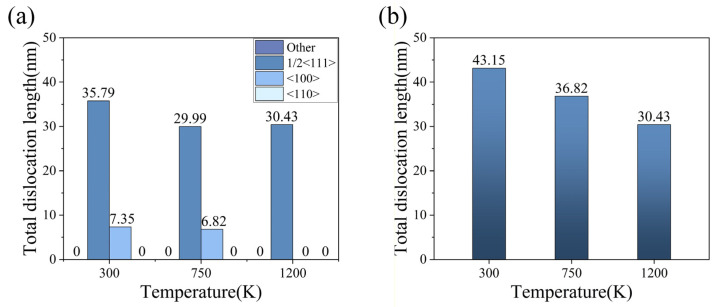
Evolution of dislocation line length under different temperature conditions: (**a**) dislocation type distribution and (**b**) total dislocation line length.

In Figure 9a, 1/2<111> dislocations dominate under all temperature conditions, while the proportion of <100> dislocations remains relatively low. At 300 K, in addition to a large number of 1/2<111> dislocations, a certain number of <100> dislocations can also be observed, with a total length of about 7.35 nm. This indicates that, under low-temperature conditions, secondary slip systems also participate in local plastic deformation. When the temperature increases to 750 K, the length of <100> dislocations decreases to 6.82 nm, suggesting that the participation of secondary slip systems becomes weaker. At 1200 K, <100> dislocations almost disappear and only the dominant 1/2<111> dislocations remain. This indicates that plastic deformation under high-temperature conditions tends to occur mainly along the primary slip systems, which helps reduce interactions between different slip systems and promote more stable plastic flow.

Figure 9b shows the statistical results of the total dislocation line length under different temperature conditions. As the temperature increases from 300 K to 750 K and then to 1200 K, the total dislocation line length decreases from 43.15 nm to 36.82 nm and 30.43 nm, corresponding to reductions of about 14.7% and 29.5%, respectively. This indicates that increasing temperature can effectively suppress dislocation multiplication and retention. At 300 K, the relatively low temperature limits atomic diffusion, making it difficult for dislocations to be released through thermal activation during deformation. As a result, longer dislocation structures are retained inside the material. With increasing temperature, thermal activation becomes stronger and dislocation slip and annihilation occur more easily, allowing some of the dislocations to be released during deformation and thereby reducing the total dislocation line length.

In addition, the enhanced mobility of dislocations at elevated temperatures increases the probability of dislocation interactions and annihilation, thereby promoting dynamic recovery processes. Meanwhile, the gradual disappearance of <100> dislocations and the dominance of 1/2<111> dislocations indicate that plastic deformation becomes increasingly governed by the principal BCC slip systems. As a result, plastic flow becomes more coordinated and defect accumulation is effectively suppressed.

To further quantify the dislocation evolution behavior under different temperature conditions, dislocation density was introduced as a supplementary indicator based on the analyses of dislocation morphology and total dislocation length. Dislocation density reflects the degree of defect accumulation per unit volume and can be used to characterize the evolution of plastic deformation during spinning. According to dislocation theory, the dislocation density is defined as the total dislocation line length per unit volume [40]:

(6) $$\rho =\frac{\sum L}{V}$$

where ρ is the dislocation density, ∑L is the total length of the dislocation line, and V is the volume.

**Figure 10:**
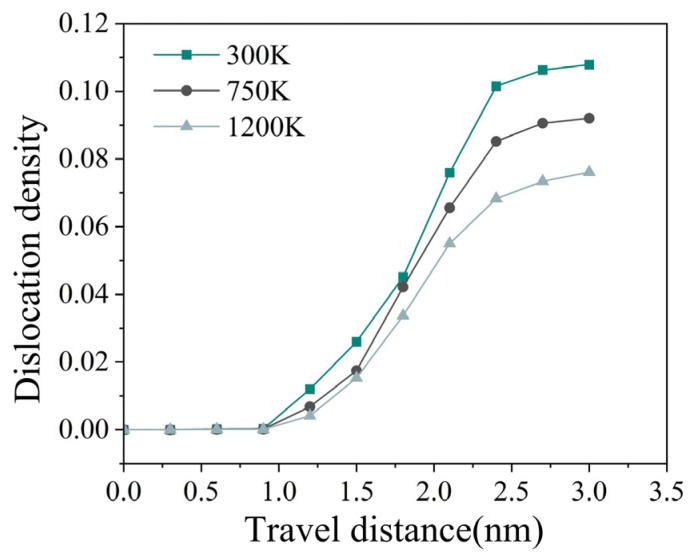
Evolution of dislocation density with sliding distance under different temperature conditions.

As shown in Figure 10, the dislocation density increases continuously with sliding distance under all temperature conditions, indicating the progressive nucleation and multiplication of dislocations during spinning deformation. Meanwhile, a significant temperature dependence can be observed. The dislocation density is highest at 300 K and lowest at 1200 K. At a sliding distance of 3.0 nm, the dislocation densities at 300 K, 750 K, and 1200 K reach approximately 0.108, 0.092, and 0.076, respectively.

The overall decrease in dislocation density with increasing temperature indicates that defect accumulation within the contact zone is effectively suppressed at elevated temperatures. Furthermore, the evolution of dislocation density is consistent with the trends observed in the dislocation morphology and total dislocation length analyses, further confirming that increasing temperature reduces dislocation retention and defect accumulation. Consequently, a lower dislocation density promotes more uniform plastic deformation and improved deformation compatibility during spinning.

## 4. Experimental Results

**Figure 11:**
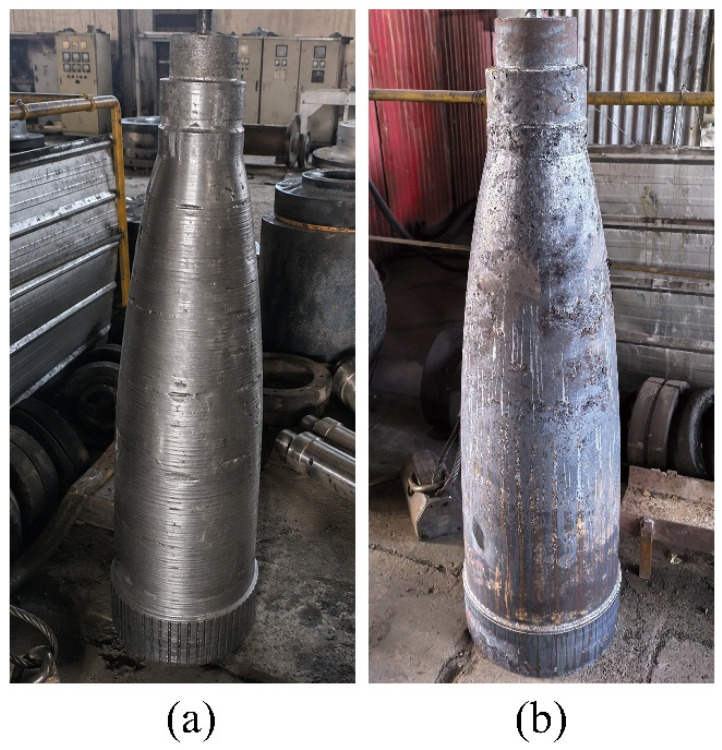
Macroscopic forming morphologies of hot-spun workpieces under different temperature conditions: (**a**) 750 K and (**b**) 1200 K.

Based on the influence of temperature on the plastic deformation and material flow behavior within the spinning contact zone revealed by the previous molecular dynamics simulations, hot-spinning experiments were conducted under different temperature conditions. Combined with the macroscopic formation quality and three-dimensional surface morphological characterization results of the spun workpieces, the material flow state and surface formation characteristics were systematically analyzed. Figure 11 presents the macroscopic morphologies of the workpieces after spinning under different temperature conditions.

As shown in Figure 11a, the workpiece surface exhibits relatively high roughness, accompanied by obvious longitudinal flow marks and localized pit defects. This behavior is mainly attributed to the insufficient plasticity of the material under low-temperature conditions. Under the localized compressive action of the spinning roller, the material cannot undergo uniform plastic flow in a timely manner, resulting in stress concentration and deformation incompatibility within the contact zone. Consequently, pronounced plastic extrusion traces and surface damage are formed on the workpiece surface. In contrast, the hot-spun workpiece shown in Figure 11b exhibits significantly improved overall surface quality, with a more uniform surface profile and more continuous material flow traces. This indicates that the deformation resistance of the material gradually decreases with increasing temperature, which helps alleviate local stress concentration within the spinning contact zone and promotes stable material flow along both the axial and circumferential directions during the spinning process. The experimental observations exhibit consistent qualitative trends with the molecular dynamics simulation results presented previously. Under elevated-temperature conditions, atoms within the contact zone can undergo rearrangement and slip more rapidly, enabling the material to be transported more continuously along the roller movement direction. As a result, local stress accumulation and material tearing phenomena are effectively reduced. Although the strain rates employed in molecular dynamics simulations are significantly higher than those in practical spinning experiments, the simulations remain effective for revealing the fundamental atomistic mechanisms underlying the experimentally observed temperature-dependent trends.

**Figure 12:**
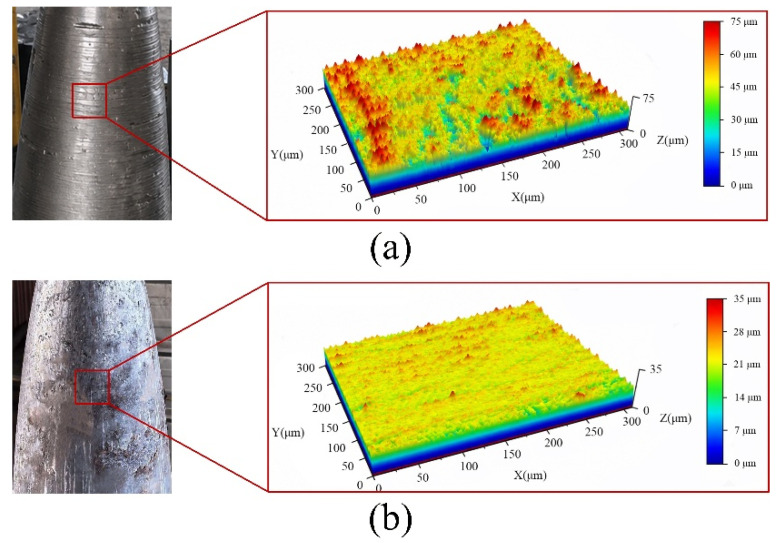
Three-dimensional surface morphologies of workpieces under different temperature conditions: (**a**) 750 K and (**b**) 1200 K.

To further investigate the surface microscopic morphological characteristics of the workpieces under different temperature conditions, a laser scanning confocal microscope was employed to perform three-dimensional surface roughness characterization. The corresponding results are presented in Figure 12. Figure 12a presents the three-dimensional surface morphology of the workpiece under low-temperature conditions. Pronounced peak–valley fluctuations can be observed on the surface, accompanied by relatively deep grooves and localized material accumulation in certain regions, indicating significant surface height variation. The color distribution contains a large proportion of red and yellow areas, suggesting prominent surface peaks and non-uniform local plastic flow. This behavior is mainly attributed to the limited material flow capability at low temperatures. During the spinning roller extrusion process, the localized material cannot spread uniformly, resulting in the formation of obvious surface waviness and pit-like structures.

In contrast, the workpiece surface under high-temperature conditions shown in Figure 12b is considerably smoother overall, with a significantly reduced peak–valley difference and a more uniform three-dimensional surface profile. This indicates that the material can undergo more sufficient plastic flow at elevated temperatures. Owing to the enhanced thermal softening effect under high-temperature conditions, local deformation during the spinning process becomes more coordinated, allowing the material to flow continuously and stably under the action of the spinning roller. Consequently, surface stress concentration and localized material accumulation are effectively alleviated.

**Figure 13:**
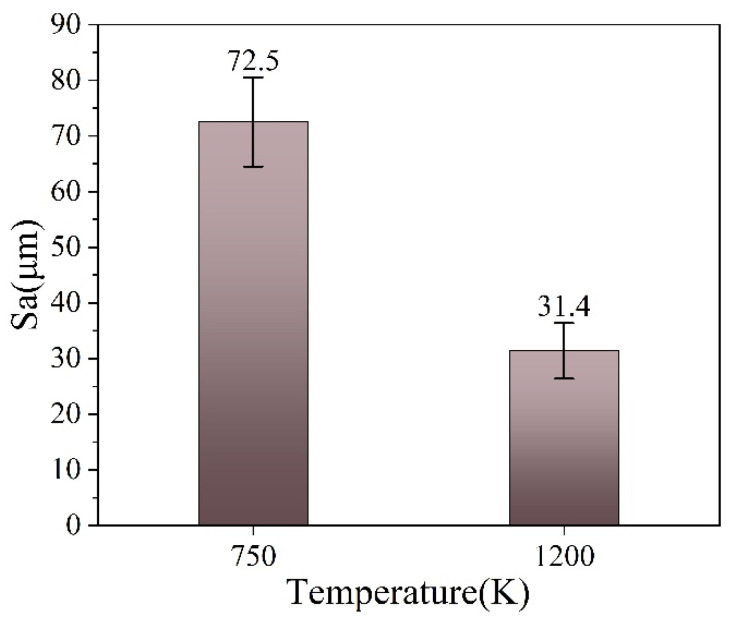
Variation in workpiece surface roughness Sa under different temperature conditions.

Figure 13 further presents the variation in the arithmetic mean surface roughness (Sa) of the workpieces under different temperature conditions. The results indicate that when the temperature increases from 750 K to 1200 K, the Sa value decreases from 72.5 μm to 31.4 μm, corresponding to a reduction of approximately 56.7%. These results demonstrate that increasing the spinning temperature can significantly improve the surface quality of the workpiece. The error bars represent the standard deviation of three repeated measurements.

Combined with the molecular dynamics simulation results, it can be concluded that elevated temperatures enhance atomic thermal vibration and improve dislocation slip activity as well as the plastic deformation coordination capability within the crystal structure, resulting in more uniform shear deformation within the contact zone. Meanwhile, the interfacial friction resistance and normal load are reduced, which alleviates local material tearing and surface accumulation phenomena. Consequently, the surface roughness of the workpiece is significantly decreased. These findings indicate that temperature not only affects the macroscopic forming load, but also governs the material flow behavior and surface formation mechanism within the spinning contact zone.

## 5. Conclusions

This study investigated microscopic deformation behavior within the spinning contact zone under different process parameters and temperature conditions using molecular dynamics simulations. The effects of indenter arc radius, indentation depth, tangential sliding velocity, and temperature on stress transfer, shear deformation, material flow, and dislocation evolution were analyzed. The main conclusions are summarized as follows:

(1) The analyses of Von Mises stress and shear strain indicate that process parameters significantly influence stress transfer and shear deformation behavior within the spinning contact zone. Large normal loading and high-speed sliding tend to induce obvious local stress accumulation and shear intensification near the contact front, whereas moderate compression–shear coupling promotes uniform stress diffusion and stable shear deformation propagation.

(2) Atomic displacement analysis shows that increasing normal compression and tangential sliding enlarges the atomic migration region and promotes obvious plastic flow along the material flow direction. Under large-indentation-depth and high-sliding-velocity conditions, pronounced displacement accumulation and local material pile-up are likely to occur near the outlet region, thereby aggravating flow non-uniformity.

(3) The analyses of normal and tangential forces indicate that the normal load during spinning is mainly controlled by indenter indentation, while the tangential load continuously increases with material shear transport. Both the normal and tangential forces decrease significantly with increasing temperature, indicating that elevated temperature can effectively reduce deformation resistance and interfacial shear resistance within the contact zone.

(4) The dislocation evolution results demonstrate that plastic deformation is dominated by 1/2<111> dislocations under all temperature conditions. As the temperature increases, the total dislocation line length decreases from 43.15 nm to 36.82 nm and 30.43 nm, corresponding to reductions of approximately 14.7% and 29.5%, respectively. Meanwhile, the <100> secondary dislocations gradually decrease and eventually disappear, indicating that elevated temperature can effectively suppress dislocation accumulation.

(5) Based on the combined experimental results and molecular dynamics analysis, it can be concluded that increasing temperature can effectively enhance the plastic flow capability of the material during the spinning process, alleviate local stress concentration and surface damage behavior, and consequently improve the overall surface formation quality of the workpiece.

## Figures and Tables

**Table 1:** Simulation parameter settings.

Simulation Parameters	Name/Value
Workpiece material	α-Fe
Indenter material	Diamond
Dimensions of workpiece	200 × 50 × 40 Å^3^
Normal indentation depth	6 Å, 8 Å, 10 Å, 12 Å
Tangential sliding velocity	1 Å/ps, 1.5 Å/ps, 2 Å/ps, 2.5 Å/ps
Indenter arc radius	15 Å, 20 Å, 25 Å, 30 Å
Relaxation temperature	293 K
Time step	1 fs

**Table 2:** Morse potential function parameters.

	$D_{0}$ (eV)	α (Å^−1^)	$r_{0}$ (Å)
Fe-C	0.4261	3.0193	2.3554
C-C	2.423	2.555	2.522

**Table 3:** Dimensional parameters of the workpiece.

Parameter	Parameter	Parameter	Parameter
Dimensions (mm)	Φ295	Φ310	Φ1520

## Data Availability

The data cannot be made publicly available upon publication because they contain commercially sensitive information. The data that support the findings of this study are available upon reasonable request from the authors.
